# Evaluation of Fusarium Head Blight Resistance in 410 Chinese Wheat Cultivars Selected for Their Climate Conditions and Ecological Niche Using Natural Infection Across Three Distinct Experimental Sites

**DOI:** 10.3389/fpls.2022.916282

**Published:** 2022-05-25

**Authors:** Zhen Yan, Wanquan Chen, Theo van der Lee, Cees Waalwijk, Anne D. van Diepeningen, Jie Feng, Hao Zhang, Taiguo Liu

**Affiliations:** ^1^College of Plant Protection, Gansu Agricultural University, Lanzhou, China; ^2^State Key Laboratory for Biology of Plant Diseases and Insect Pests, Institute of Plant Protection, Chinese Academy of Agricultural Sciences, Beijing, China; ^3^Institute of Pomology, Chinese Academy of Agricultural Sciences, Xingcheng, China; ^4^National Agricultural Experimental Station for Plant Protection, Gangu, Ministry of Agriculture and Rural Affairs, Tianshui, China; ^5^Wageningen University and Research Center, Wageningen, Netherlands

**Keywords:** fusarium head blight, resistance, mycotoxin, multi-locations, different years

## Abstract

Exploiting wheat cultivars with stable resistance to Fusarium Head blight (FHB) and toxin accumulation is a cost-effective and environmentally friendly strategy to reduce the risk of yield losses and contamination with mycotoxins. To facilitate the deployment of stable cultivar resistance, we evaluated FHB resistance and resistance to mycotoxin accumulation in 410 wheat lines bred by local breeders from four major wheat growing regions in China after natural infection at three distinct locations (Hefei, Yangzhou and Nanping). Significant differences in disease index were observed among the three locations. The disease indexes (DI’s) in Nanping were the highest, followed by Yangzhou and Hefei. The distribution of DI’s in Yangzhou showed the best discrimination of FHB resistance in cultivars. Growing region and cultivar had significant effect on DI and mycotoxins. Among the climate factors, relative humidity and rainfall were the key factors resulting in the severe disease. Even though most cultivars were still susceptible to FHB under the strongly conducive conditions applied, the ratio of resistant lines increased in the Upper region of the Yangtze River (UYR) and the Middle and Lower Region of the Yangtze River (MLYR) between 2015 and 2019. Deoxynivalenol (DON) was the dominant mycotoxin found in Hefei and Yangzhou, while NIV was predominant in Nanping. Disease indexes were significantly correlated with DON content in wheat grain.

## Introduction

Fusarium head blight (FHB) caused by members of the *Fusarium graminearum* species complex (FGSC) is a devastating disease of wheat in China and many other regions of the world (Bai and Shaner, 2004; van der Lee et al., 2015; Buerstmayr et al., 2020). FHB epidemics occur frequently in the Yangtze River Region (Chen et al., 2000). However, during the last decades, FHB epidemics were also prevalent in the north of the Huai valley and the southern part of the Huanghuai valley, where previously FHB epidemics were rare (Zhang et al., 2012). FHB not only causes severe losses in both yield and quality, but, more seriously, the toxins produced during FHB infection pose a serious health threat to humans and animals (Ma et al., 2019; Buerstmayr et al., 2020), The most frequently encountered mycotoxins in China are deoxynivalenol (DON) and nivalenol (NIV; Yang et al., 2018; Yerkovich et al., 2020).

Breeding and planting wheat cultivars with stable resistance to FHB and toxin accumulation is considered to be the most cost-effective and environmentally friendly strategy to reduce the risk of yield losses and contamination with mycotoxins (Bertuzzi et al., 2020; Maré et al., 2020). Different resistance mechanisms to FHB have been described in wheat (Mesterházy et al., 1995; Mesterházy, 2020). The first two mechanisms of resistance are related to severity and include Type I: resistance to initial infection, and Type II: resistance to spread to other spikelets. Type III and IV are related to post-harvest traits, e.g., DON accumulation and *Fusarium* damaged kernels (FDK) and *Fusarium* colonized kernels (FCK) respectively, while type V refers to tolerance to infections (Spanic et al., 2019; Mesterházy, 2020). Mycotoxin content, grain yield losses and impact of infection on seed quality are undoubtedly the most important characters (He et al., 2019). In China more than 70 years of wheat breeding for resistance to FHB resulted in moderately resistant and resistant cultivars. Despite the existence of these FHB resistant cultivars, they are not always selected (by farmers) and currently more than 90% of prevailing wheat cultivars are still susceptible (Dong et al., 2020).

Many studies estimated the resistance level of wheat cultivars to FHB and mycotoxin contamination (e.g., Tamburic-Ilincic, 2012; Lemmens et al., 2016). In most of these studies FHB resistance evaluation was performed at only a single site. This can be problematic as for field experiments, environmental factors are important, temperature and rainfall are key climatic factors that have large impact on FHB severity and mycotoxins concentrations. When identical cultivars are evaluated by different research institutions at different locations or in different years’ results may be inconsistent, limiting the value of testing all cultivars at one location for advice to growers. Only very few reports of multiple locations and multiple years are available under natural infections. Groth et al. (2011) reported on the comparison of 40 wheat cultivars for FHB index and DON concentrations in multi-environmental field trials in Canada and Germany. Canadian and European wheat lines were shown to be stable in performance across both countries. In contrast, Lu et al. (2002) evaluated FHB resistance of two wheat genetic populations (305 lines) in 1999 and 2000 at four sites, which showed a poor correlation of individual FHB resistance either from different places in 1 year or from a single location at different years. In addition, He et al. (2019) analyzed FHB and associated traits including DON accumulation for 197 lines in field experiments at CIMMYT (Mexico) across 4 years, which showed that FHB disease pressure varied greatly among years, and “year” was the major source of variation for both FHB severity and DON accumulation. The above reports were based on artificial inoculation with a focus on Type II resistance, while a natural infection may be more informative for the selection of FHB resistant cultivars by growers.

In 2015, we evaluated the resistance level and mycotoxins accumulation of 129 wheat cultivars after natural infection (Yan et al., 2020), performed in a single nursery and the stability of the FHB resistance across different environments/places remained unclear. To circumpass these drawbacks, in this study we evaluated 410 wheat cultivars recently bred by local breeders in four ecological regions across China at three locations with different climatic conditions. The main scientific aims were: (i) to compare the stability of resistance evaluation of FHB at these three locations under natural infection; (ii) to discuss the relative importance of region, cultivars and climate factors in affecting the severity of FHB; and (iii) to investigate the correlation between toxin accumulation and FHB disease resistance among wheat cultivars from four ecological regions. This designed multi-locations evaluation provides a new standard to improve the accuracy and stability of FHB resistance evaluation, contributes to the current breeding programs and the deployment of resistant cultivars by growers.

## Materials and Methods

### Field Trials

**Figure 1:**
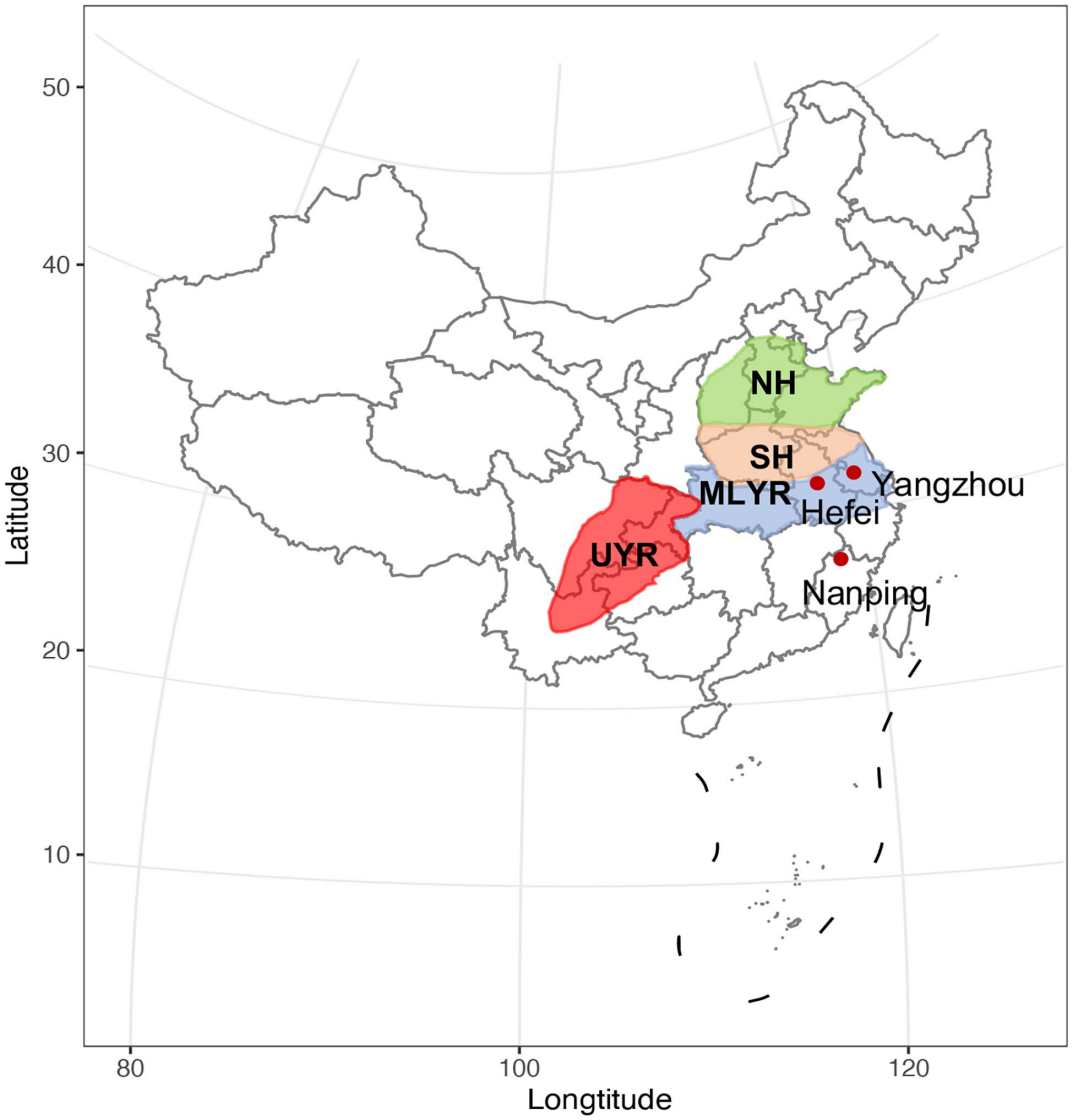
The four ecological regions of wheat production in China. UYR, the upper reaches of Yangtze River Region; MLYR, the middle and lower reaches of Yangtze River Region; SH, the south of the Huanghuai Region; NH, the north of the Huanghuai Region. The three nurseries are indicated with red dots.

A total of 410 wheat cultivars which were ready for national cultivar registration (2019) were evaluated in this study. They were newly bred by local breeders in four major wheat growing regions in China (Figure 1): (1) the upper reaches of Yangtze River Region (UYR, *n* = 32), including Sichuan, Yunnan and Chongqing provinces, where the climate is wet and warm, classified as a moderate FHB epidemic area. In this region, wheat is rotated with either rice or maize, the ratio of planting acreage (wheat/rice or maize) is about 3:1; (2) the middle and lower reaches of Yangtze River Region (MLYR, *n* = 60), including Hubei, Zhejiang and the southern parts of Anhui and Jiangsu provinces, where the climate is wet and warm, usually rainy during wheat flowering stage, and considered a high risk FHB epidemic area. Wheat/rice rotation is the predominant cropping system in this region; (3) the southern part of the Huanghuai Region (SH, *n* = 273), including Henan, Shanxi, the northern parts of Anhui and Jiangsu, and the southern part of Shandong, where the climate is warm and dry, rated as moderate FHB epidemic risk areas; and (4) the north part of the Huanghuai Region (NH, *n* = 45), including Hebei and the northern part of Shandong, where the climate is cool and dry, these regions are considered mild FHB epidemic risk areas. Wheat/maize rotation dominate both the north and south parts of the Huanghuai Region, is especially suitable for the survival and propagation of *F. graminearum*.

All of the 410 wheat cultivars were tested at three National Wheat FHB Resistance Evaluation Nursery in 2019, including Nanping (27°43′ north latitude, 118°12′ east longitude) in Fujian province, Yangzhou (32°24′ north latitude, 119°26′ east longitude) in Jiangsu province and Hefei (31°49′ north latitude, 117°13′ east longitude) in Anhui province. Yangzhou and Hefei, located in eastern China, have a similar level of altitude and climate. They have experienced large challenges due to FHB incidence and contamination with *Fusarium* mycotoxins in the last decades, due to rotation and warm weather during the heading, flowering and filling stage of wheat growth (Dong et al., 2016; Xu et al., 2019). The two regions are traditional FHB epidemic areas in the lower reaches of the Yangtze River. Nanping, located further south in China, is rainy and humid throughout the wheat growing season, leading to the severe and stable occurrence of FHB in this area all year round. Farmers abandoned wheat cultivation due to severe FHB epidemics more than 20 years ago. Therefore, the environmental conditions in Nanping, Hefei, and Yangzhou provide a unique condition for the natural occurrence of FHB in the field. In view of this, these three regions were selected as the test sites for resistance evaluation of FHB by natural infection.

The cultivars were arranged in a randomized block design in single rows (length 100 cm, line spacing 25–33 cm) which contained approximately 300 plants in one line, with three replicates for each cultivar. No fungicides were applied on the experimental plot. The local daily temperature, relative humidity, sunshine duration, soil surface temperature and accumulated rainfall at each location were recorded by a weather station in the field, the comparison of these climate factors during the disease related period (from 10 April to 23 May in Heifei and Yangzhou, and from 16 March to 28 April in Nanping) among the locations were performed by generalized linear model (GLM).

### Evaluation of Resistance by Fixed DI Ranges

The incidence of FHB was recorded at the late milky stage. Disease severity was scored on ten random wheat ears per plot, in total 30 wheat ears were investigated per cultivar. Disease severity was estimated visually *in situ* on a 0–4 scale: 0, no visible symptoms; 1, < 25% affected spikelets; 2, 25–50% affected spikelets; 3, 50–75% affected spikelets; and 4, > 75% affected spikelets. The disease index was calculated using Eq. (1), where *X_i_* means the number of infected spikelets, *S_i_* means the scale of disease severity (0–4), *X*_max_ means the numbers of ears scored (30), and *S*_max_ stands for the maximum disease severity (4):


(1) $$\mathrm{Disease index}\;\left(\%\right)=\overset{n}{\underset{i=0}{\sum }}\frac{X_{i}\times S_{i}}{X_{\max}\times S_{\max}}\times 100.$$

The resistant level was graded by disease index: highly resistant (HR) = DI below 10; moderately resistant (MR) = DI between 10 and 25; moderately susceptible (MS) = DI between 25 and 45; highly susceptible (HS) = DI higher than 45 (Yan et al., 2020).

### Evaluation Method for Consistency of Resistance to FHB Between Three Nurseries

Because the disease severity is affected greatly by local climatic conditions, the resistant level of cultivars between three nurseries cannot be simply compared using the absolute disease index (DI) values. Therefore, the DI of wheat cultivars in each nursery (Hefei, Yangzhou and Nanping) was classified into four resistant groups according to the quartile method (A: 0–25% of cultivars; B: 25–50% of cultivars; C: 50–75% of cultivars; D: 75–100% of cultivars) to represent the distribution of the DI in each nursery. A pairwise Fisher’s exact test was used to compare the proportion of each group of wheat cultivars from the four ecological regions. The correlation of DIs between Yangzhou and Nanping nursery was determined using generalized linear model (GLM) and Spearman’s correlation coefficient.

### Wheat Grain Sample Pretreatment

At harvest time, all wheat ears were collected manually for toxin determination from each plot. They were threshed with a laboratory thresher at low wind speed to prevent loss of low-weight infected kernels. Each sample was further milled to a fine powder with the IKA Tube Mill (IKA-Werke, Germany). Five gram samples were supplemented with 25 ml of 80% (*v*/*v*) acetonitrile, shaken for 2 min and then centrifuged at room temperature for 3 min at 9,000 rpm. Afterwards, 2 ml of supernatant was sequentially purified by passing through the multifunctional purification column (Bond Elut Mycotoxin, Agilent, United States) and through an 0.22 μm nylon filter for UPLC-MS/MS analysis.

### Reagents and Standards

Acetonitrile and methanol (MSgrade) were purchased from Merck KGaA (Darmstadt, Germany). Deionized water (18 MΩ*cm) was produced in the laboratory using a Milli-Q Reagent Water System (Millipore, United States). The standards of DON and NIV were purchased from Pribolab Pte. Ltd. (Pribolab, Singapore).

### UPLC-MS/MS Method

UPLC-MS/MS was performed on a Micromass QuattroUltima triple-quadrupole mass spectrometer equipped with an ESI source (Xevo TQS, Waters, United States). Chromatography was performed on a reversed phase UPLC BEH C18 column (2.1 × 100 mm, 1.7 μm) from Waters Scientific. Mobile phases were methanol (A) and 4 mmol/l of ammonium acetate (B). The gradient program was as follows: 0–2 min, 10–90% A; 2–4 min, 90% A; 4–4.1 min, 90–10% A; 4.1–6 min, 10% A. The injection volume was 2 μl with a flow rate of 0.3 ml/min and the column temperature was maintained at 40°C. The instrument featured an ESI source and was operated in both positive and negative ion modes. Quantitation was performed using MRM. The source temperature was 150°C, and the desolvation gas temperature was 350°C. The desolvation gas and nebulizer gas (N_2_) were set at 650 and 50 l/h, respectively. The flow rate of the collision gas (Ar) was 0.14 ml/min.

### Statistical Analysis

The correlation effect of cultivar and growing region on severity and mycotoxin levels was calculated by generalized linear model (GLM). All data analysis was performed using R Software.

### Comparison of FHB Resistance Levels in 2015 and 2019

We previously reported the resistance to FHB in 129 wheat cultivars from different ecological regions planted in Nanping in 2015. These samples were also bred by local breeders and submitted for national approval. To briefly investigate a potential change in resistance level on a national scale, we compared the resistance levels of wheat cultivars to FHB in 2015 and 2019.

The DI of the highly susceptible control cultivar Zhoumai 18 can reflect the discrimination level of the evaluation. Because the DI of Zhoumai 18 in Nanping_2015 was lower than in Yangzhou_2019, the DI of all cultivars in 2015 was normalized using Eq. (2) before comparison, where DI_1_ means the original disease index, DI_2_ means the corrected disease index. DI_CK1_ and DI_CK2_ means the disease index of Zhoumai 18 in 2015 and 2019, respectively.


(2) $${\mathrm{DI}}_{2}=\frac{{\mathrm{DI}}_{\mathrm{CK2}}*{\mathrm{DI}}_{1}}{{\mathrm{DI}}_{\mathrm{CK1}}}.$$

## Results

### Fusarium Head Blight Severity in Different Nurseries

**Figure 2:**
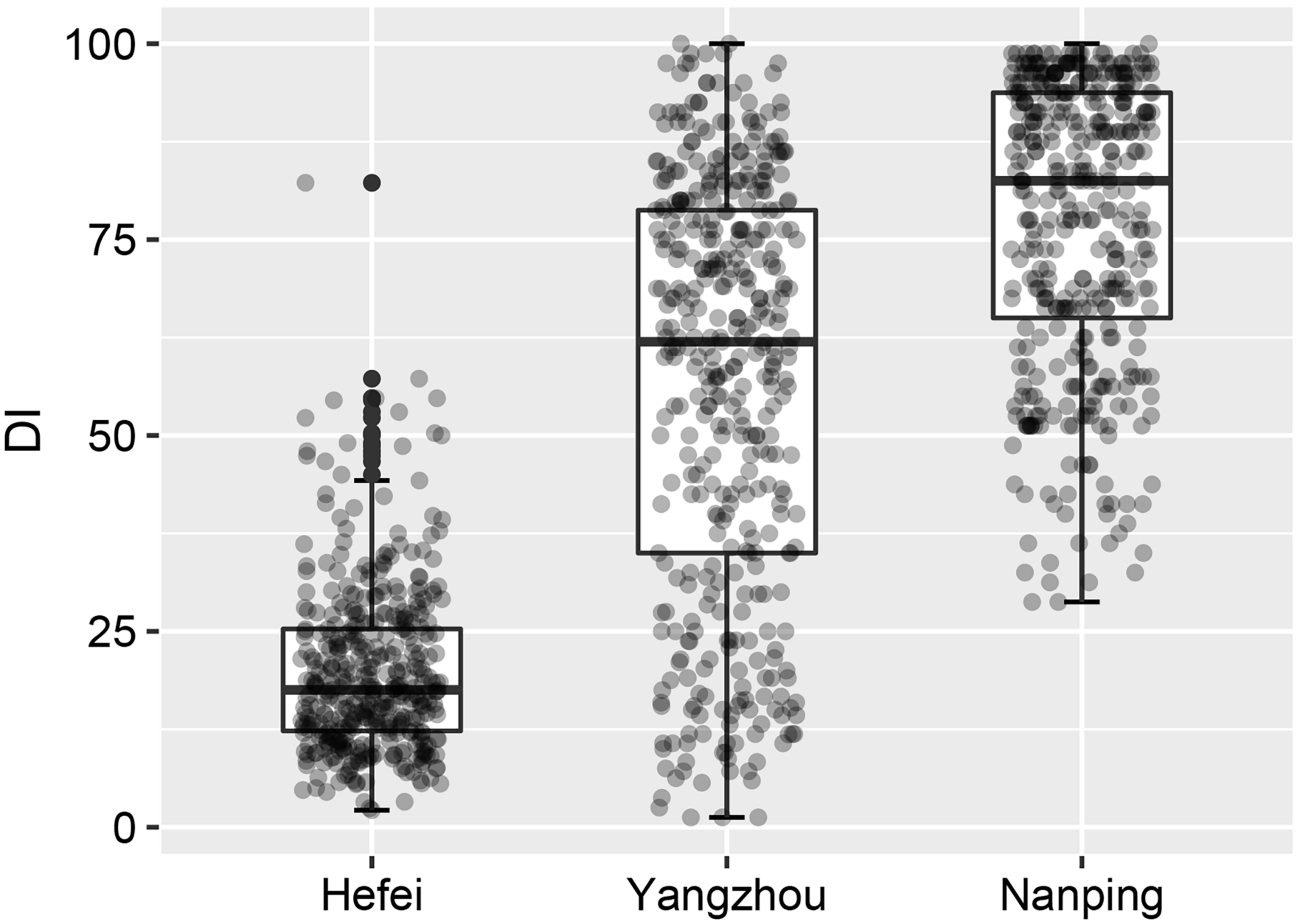
Boxplots of the disease indexes of 410 wheat cultivars in three nurseries, demonstrating that the disease pressure in Hefei was much lower than in the other two locations.

The FHB severity of 410 wheat cultivars at three nurseries Hefei, Yangzhou and Nanping were compared. The results are shown in Figure 2. Significant differences in disease index (DI) were observed among the three experimental sites (*p* < 0.001). In Hefei, the severity of FHB was the lowest with the median DI of 17.5. The lowest interquartile range (IQR; 13.0) indicated the lower ability to discriminate FHB resistance in Hefei. In Nanping the disease was the most severe, with a median DI of 82.5. The IQR was 28.8, which is much wider than in Hefei, indicating a better discrimination between cultivars/accessions. The disease index in Yangzhou was intermediate with a median DI of 62.0. The widest IQR (43.8) was observed in Yangzhou. The wide and even distribution of DI revealed the best discrimination of differences in FHB resistance of the cultivars can be obtained in Yangzhou. The disease index of 410 cultivars are summarized in Supplementary Table S1.

### Comparison of the FHB Resistance Composition of Cultivars Based on the Quartile Method Between Three Nurseries With Different Disease Severity Levels

**Figure 3:**
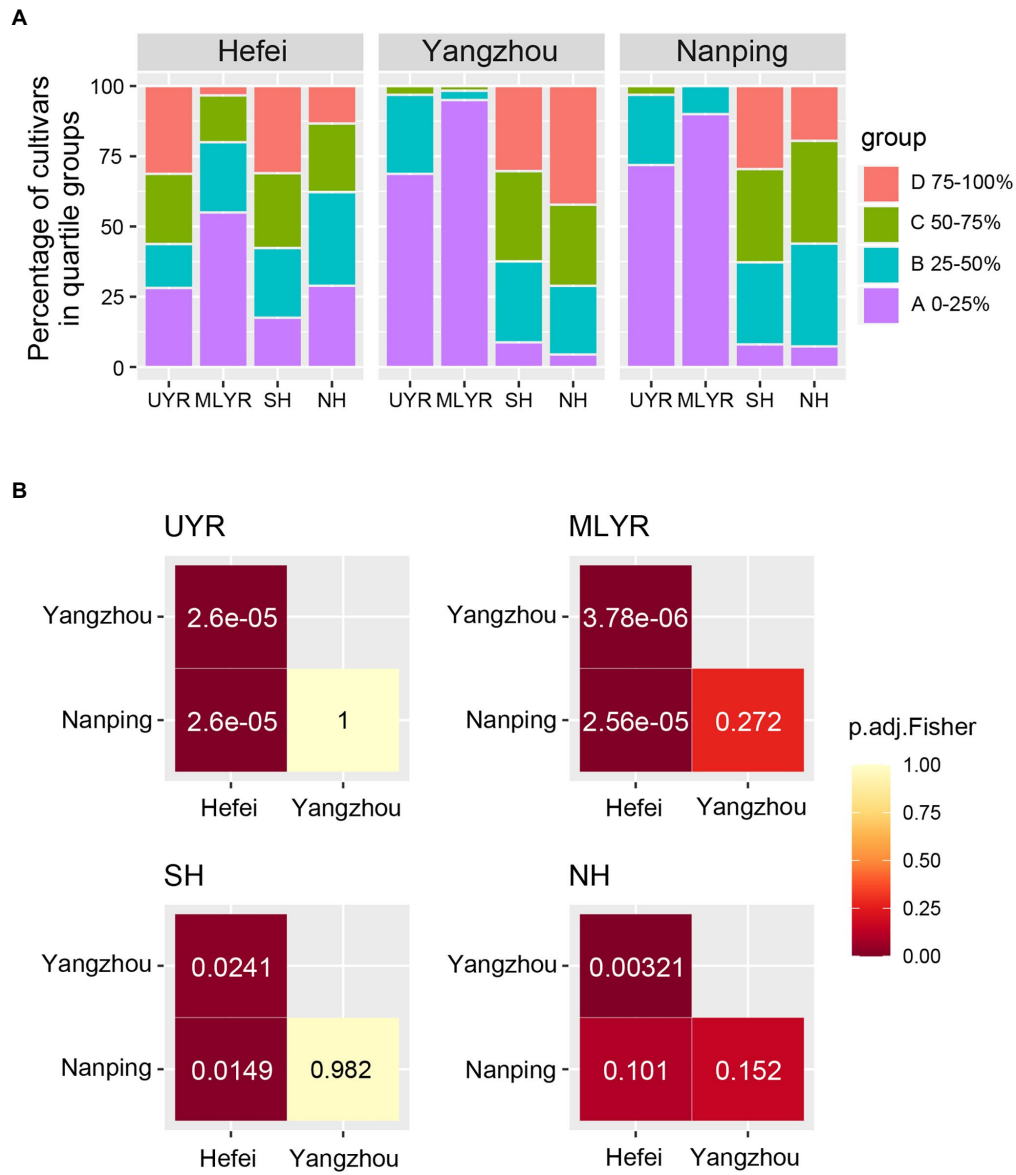
Resistant composition based on quartile method for wheat cultivars from four ecological regions between three nurseries. **(A)** Distribution of quartile groups in each of the three nurseries (A: 0–25% of cultivars; B: 25–50% of cultivars; C: 50–75% of cultivars; D: 75–100% of cultivars); **(B)** Fisher’s exact test of composition of quartile groups between three nurseries.

The resistance composition of cultivars originating from ecological regions (UYR, MLYR, SH, and NH) is shown in Figure 3A. Yangzhou and Nanping showed a similar distribution pattern of resistance composition for cultivars from each of the four ecological regions. Cultivars with higher FHB resistance (group A) dominated MLYR (95% in Yangzhou, 90% in Nanping). Among cultivars from UYR, group A was also the predominant resistance level (69% in Yangzhou, 72% in Nanping), followed by group B with moderate FHB resistance (28% in Yangzhou, 25% in Nanping). Cultivars from SH and NH mainly belong to group B and two susceptible groups C and D, the percentage of the three groups were similar. However, the distribution across resistance composition in Hefei was very different. Cultivars from each ecological region distribute across all four resistant groups and there were no clear compositional differences among the regions with the sole exception of a large proportion of group A cultivars in MLYR (55%).

A pairwise Fisher’s exact test was performed for each wheat originating ecological region to further evaluate the consistency of FHB resistance evaluation in different nurseries (Figure 3B). There was no significant difference on the resistance composition between Yangzhou and Nanping in all four ecological regions, and the *p* values ranged from 0.152 to 1. This indicated good consistency between the two nurseries. However, the resistance composition of each ecological region tested in Hefei showed significant differences (*p* < 0.05) with the corresponding regions in Yangzhou and Nanping. The only exception were the cultivars from the NH region, which showed a *p* value of 0.101 between the Hefei and Nanping nurseries. This suggested the mild disease pressure in Hefei cannot truly reflect the resistant level of the cultivars.

**Figure 4:**
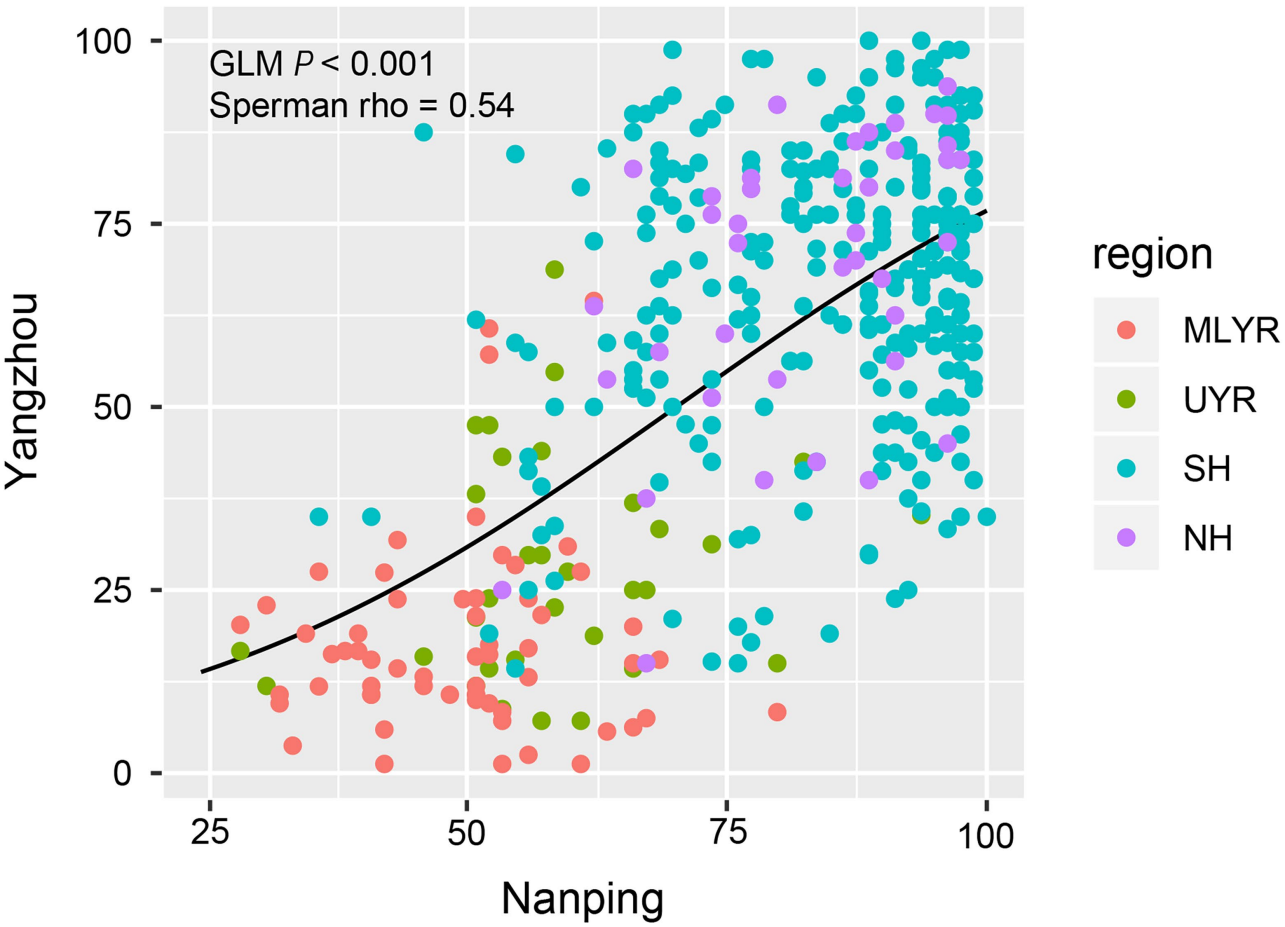
The correlation of Fusarium Head Blight DIs for 410 cultivars between the nurseries in Nanping and Yangzhou (x and y axes represent the DI value). The cultivars from MLYR showed low DIs in both locations, followed by the cultivars from UYR. The majority of cultivars from both SH and NH showed higher DIs at both nurseries. Removal of those cultivars showing DIs > 90 improved the correlation from rho = 0.54–0.64.

The two nurseries in Yangzhou and Nanping showed a very good consistency in resistance group distribution, and further analysis revealed a significant correlation of DIs for all cultivars between both locations (GLM: *p* < 0.001; Spearman’s correlation: *p* < 0.001, rho = 0.54). The DI values of the majority of cultivars in Yangzhou and in Nanping were similar (Figure 4). The DIs of the cultivars from MLYR were at low level, followed by the ones from UYR, while the DIs of cultivars from both Huanghuai Regions (NH and SH) were relatively high. However, in Nanping we found 148 cultivars (1 from UYR, 134 from SH, 13 from NH) with a high DI (DI ≥ 90) compared to only 34 cultivars in Yangzhou (Figure 4). If we remove these 148 cultivars, the Spearman’s rank correlation coefficient (rho) increased to 0.64. Although the resistance composition between Yangzhou and Nanping was consistent on the whole (Figure 3), there was a large number of cultivars (36%) in Nanping with high severity in each sample (Figure 4).

### Mycotoxins Accumulation of Wheat Cultivars From Different Ecological Regions

Due to severe disease, not enough grains or no grains could be harvested for mycotoxin determination from some cultivars. Especially in Nanping, 56% of the cultivars could not be used for mycotoxin analysis, including most cultivars originating from SH and NH. As a consequence, the number of cultivars involved in the subsequent mycotoxin determination was 408 in Hefei, 387 in Yangzhou and only 181 in Nanping. The toxin contents are summarized in Supplementary Table S1.

DON was detected in all samples. The DON concentration in 387 cultivars in Yangzhou was the highest with a mean of 12.87 mg/kg, followed by Nanping with a mean of 2.39 mg/kg and Hefei with a mean of 1.13 mg/kg (Table 1). DON dominated in both Yangzhou and Hefei, while the main mycotoxin contamination in Nanping was NIV, the mean value of NIV content (7.63 mg/kg) was about three times more than DON (2.39 mg/kg).

**Table 1:** The concentration of mycotoxins in the three ecological regions.

Regions	Toxins	Min (mg/kg)	Max (mg/kg)	Mean (mg/kg)	Frequency (%)^a^
Hefei	DON	0.011	4.94	1.13	100
	NIV	0.003	2.68	0.59	100
Yangzhou	DON	0.21	49.4	12.87	100
	NIV	0	0.61	0.053	30.4
Nanping	DON	1.10	6.1	2.39	100
	NIV	2.77	29.0	7.63	100

a *Percentage of positive samples.*

### Relation Between Cultivar Source and Resistance to Mycotoxin Accumulation in Yangzhou and Nanping

**Figure 5:**
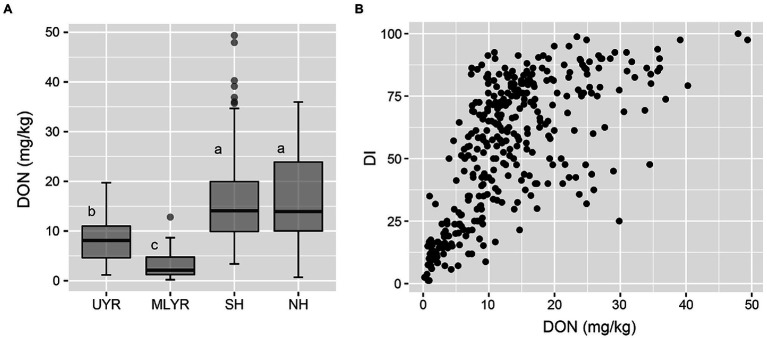
DON accumulation level of wheat cultivars from four ecology regions **(A)** and the correlation between DI and DON content **(B)** in the Yangzhou nursery.

In Yangzhou, the DON concentration of the wheat cultivars from both Huanghuai Regions were significantly higher than that from Yangtze River Region, and there was no significant difference between SH and NH (Figure 5A). Regression analysis between DI and toxin accumulation was performed by GLM. DI was significant correlated with DON in Yangzhou (*p* < 0.05; Figure 5B). In Nanping, we analyzed the correlation between the dominant mycotoxin NIV and DI of the 181 harvested cultivars. However, different from DON in Yangzhou, there was no significant difference between DI and NIV (*p* > 0.05).

### Effect of Cultivar and Growing Regions on DI and Mycotoxin Accumulation

The effects of growing regions and cultivar on severity and toxin level were evaluated by GLM. The results showed that the *p* values were all below 2.2e-16 (Table 2). This demonstrates that both region and cultivar had significant effect on DI and mycotoxins.

**Table 2:** Effect (*p*-value) of cultivars (Cvs) and nurseries (Nus) on severity and mycotoxins level.

Source	*Df*	Deviance	R.Df	R.Dev	*P* <	*Df*	Deviance	R.Df	R.Dev	*P* <
	DI					Mycotoxin				
Nus	2	15,155	1,223	10,186	2.2e-16^***^	2	3,647	800	2305.8	2.2e-16^***^
Cvs	409	6012.2	816	19,329	2.2e-16^***^	407	2080.6	395	3872.2	2.2e-16^***^
Nus x Cvs	1,225	25,341	0	0	2.2e-16^***^	802	5952.8	0	0	2.2e-16^***^

R represent resid. ***Indicates significant at p < 0.001.

### Climatical Analysis Among the Growing Regions

Significant differences in the climatical factors were observed among the three experimental sites (Table 3). In Nanping significant higher daily average relative humidity was observed, with less hours of sunshine and lower soil surface temperature compared to the other two locations. The accumulated rainfall around wheat flowering period was more than five times higher in Nanping. For Hefei and Yangzhou, located at a similar altitude, there was no significant difference in daily average temperature, sunshine duration and soil surface temperature. However, the relative humidity in Yangzhou was significant higher than Hefei and the accumulated rainfall was also 28% higher in Yangzhou.

**Table 3:** ANOVA of the climatical factors among the three nurseries.

Source	Temperature (**°**C)	Relative humidity (%)	Rainfall (mm)	Sunshine duration (h)	Soil surface temperature (**°**C)
Hefei	18.8 ± 0.6 ab	65.4 ± 2.7 c	39.8	5.1 ± 0.6 a	22.7 ± 0.7 a
Yangzhou	19.3 ± 0.5 a	72.4 ± 2.2 b	51.1	6.1 ± 0.7 a	23.2 ± 0.7 a
Nanping	17.6 ± 0.6 b	84.9 ± 1.2 a	265.5	2.5 ± 0.5 b	19.7 ± 0.7 b

### Comparison of FHB Resistance in 2015 and 2019

When the DI of cultivar Zhoumai 18 was around 50.0, the discrimination for FHB resistance provided the best resolution (50.0 in Yangzhou in 2019 and 45.7 in Nanping in 2015). If the DI was much higher (96.0 in Nanping in 2019) or much lower DI (23.0 in Hefei in 2019) the accuracy of the FHB resistance evaluation was significantly reduced (Supplementary Figure S1). Therefore, the data of Yangzhou in 2019 were selected for the comparison with the Nanping data of 2015. The DI of cultivars in the two groups were graded into four resistant levels shown in Supplementary Figure S2.

In Nanping in 2015, highly resistant, moderately resistant, moderately susceptible, and highly susceptible cultivars accounted for 1.5, 15.5, 31.8, and 51.2% of the total number of samples, respectively. Most of the wheat cultivars (83%) were susceptible to FHB, and the highly resistant cultivars were both from MLYR, accounting for 8% of the cultivars in this region. In the Yangzhou nursery in 2019, highly resistant, moderately resistant, moderately susceptible, and highly susceptible cultivars accounted for 4.2, 14.1, 13.9, and 67.8% of the total number of cultivars, respectively. Most cultivars (81.7%) were still susceptible to FHB, but the proportion of resistant cultivars has increased between 2015 and 2019. Three highly resistant cultivars were also identified in the UYR, accounting for 9.4% of the cultivars in this region. Ten and one moderately resistant cultivars were found from SH and NH, accounting for 3.7 and 2.2%, respectively, while no highly resistant cultivar was identified in cultivars originating from these two regions.

## Discussion

Different wheat cultivars have significant differences in their resistance to FHB and mycotoxin accumulation. Screening and breeding cultivars with stable resistance to FHB and toxin accumulation are the safest and most effective measures to prevent and control FHB (Bai and Shaner, 2004). Wheat breeders began to select plants or spikes with resistance to FHB in disease epidemic areas in China since the late 1950s. However, 96% of the cultivars released in China are moderately or highly susceptible to FHB, and only 4% of the cultivars released from the national cultivar trials from 2005 to 2016 had moderate resistance to FHB (Yan et al., 2020). Convincing growers to select and exploit FHB resistant cultivars is therefore important and can be demonstrated in trials. Regional environmental and climatic conditions could influence such evaluation of resistance to FHB and/or mycotoxin accumulation of wheat (Lu et al., 2002). To improve the accuracy of the evaluation, we compared FHB severity and mycotoxins accumulation of a national collection of 410 cultivars in three National Wheat FHB Resistance Evaluation Farms by natural infection. Significant differences were observed among the three locations. Wheat cultivars planted in Nanping showed the highest FHB severity, followed by Yangzhou. FHB was mildest in the nursery in Hefei. In addition, the severity of FHB of wheat is strongly driven by meteorological factors (De Wolf et al., 2003). Weather conditions during wheat flowering are the key factor for *Fusarium* infection and disease spread (Xu et al., 2021). In general, the air humidity and precipitation during flowering enhances the risk of FHB epidemics (Ji et al., 2014; Scala et al., 2016; Vaughan et al., 2016).

Nanping is the southernmost nursery among the three places and the environmental conditions are extremely conducive for FHB, as it rains very frequently from early-March to mid-April when wheat is in heading, flowering and milky stages. Because of severe FHB epidemics in this area, growers stopped wheat cultivation 20 years ago, but this nursery is still a good location for the evaluation of resistance of wheat cultivars to this disease by natural infection due to the suitable climate conditions and presence of abundant inoculum. The nurseries in Yangzhou and Hefei are at a similar latitude, and the climate is also similar in most years. Rainfall in these two places is mainly in spring and summer.

The temperature, relative humidity, rainfall, sunshine hours and soil surface temperature around wheat flowering period in the three locations are summarized in Supplementary Table S2. Significant higher relative humidity and rainfall were observed in Nanping, which would promote the disease. Lower soil surface temperature and high humidity in this place will also facilitate perithecia formation and consequently result in higher spore densities and inoculum build up for infection. Furthermore, shorter sunshine duration can reduce the UV damage of the mycelia and conidia of the pathogen. These can explain the high level of disease severity in Nanping. There was no significant difference on temperature, sunshine hours and soil surface temperature between Hefei and Yangzhou, which are at similar latitude. This indicated the significant higher relative humidity and rainfall were the key factors resulting in the more severe disease in Yangzhou compared to Hefei.

The DIs of cultivars in Yangzhou were widely and evenly distributed and the IQR was much larger than in Hefei or Nanping, revealing the best discrimination of FHB resistance. Both high and low DI would potentially reduce the accuracy of FHB evaluation. In this study, DIs of cultivars in Hefei were relatively low and distributed in a narrow range, which complicates discrimination of FHB resistance levels. Based on the quartile method, the low severity in Hefei also caused significantly inconsistence (*p* < 0.05) of the composition of FHB resistant groups with other nurseries, indicating FHB resistance evaluation with such low disease pressure is not reliable. In contrast to the results obtained in Hefei, the composition of FHB resistant groups in Nanping and Yangzhou were similar, despite an extremely high FHB severity observed in Nanping. The Fisher’s exact test revealed no significant difference in the composition between both nurseries for cultivars from each of the four ecological regions. GLM regression and Spearman’s correlation analysis also indicated significant correlation between the DI of the same cultivars at both locations. However, more cultivars distributed in high-DI quartile in Nanping (Figure 2). The increase of Spearman’s correlation coefficient after removing part of cultivars with high DI in Nanping revealed poor discrimination for susceptible cultivars in Nanping’s evaluation. The severe disease in Nanping also explained why farmers gave up wheat growing in this region and the disease pressure is still high even in absence of wheat cultivation 20 years later. Therefore, FHB evaluation in Yangzhou showed the best discrimination, which was used for subsequent analysis.

Resistant levels (HR, MR, MS, and HS) are used for description of FHB resistance in the national wheat cultivars registration. Although the different weather conditions among years would influence the evaluation result to some extent, frequency of resistant levels were still used to estimate the change of FHB resistance levels of new cultivars over the years on the national scale. Based on this strategy, we tried to investigate the change of FHB resistance of cultivars for national cultivar registration in China by comparing their frequency of resistant levels in Yangzhou_2019 and Nanping_2015, both of which showed good discrimination of FHB resistance. To improve the accuracy of the comparison, we normalize the DIs based on the susceptible control cultivar Zhoumai 18. In this study, the highest proportion of resistant cultivars were found from the Yangtze River Basin (UYR and MLYR). This is consistent with our previous report in Nanping in 2015 (Yan et al., 2020). As the climate of the Yangtze River region is humid and rainy which causes severe FHB epidemics, we proposed that growers in this region are more likely to select cultivars with higher FHB resistance. The strong selection pressures may also have provided conditions for the natural mutations of FHB resistance to be preserved in wheat. The ratio of resistant cultivars (MR + HR) increased in both UYR (from 19 to 47%) and MLYR (from 72 to 81%) from 2015 to 2019. These changes of frequency of resistant cultivars revealed the FHB resistance was improved continuously in the Yangtze River Basin in the past few years. Large changes were observed on susceptible cultivars, moderately susceptible cultivars decreased from 32 to 14% and highly susceptible cultivars increased from 51 to 68%. This is mainly due to the strong increase (three times) in the number of cultivars tested from NH and SH. Especially 77% of cultivars from SH showed increased susceptibility. Highly susceptible cultivars were predominant in SH because of the lack of local FHB resistance resources. The ratio of highly susceptible cultivars mainly increased in SH (from 72 to 85%) and NH (from 80 to 87%). Although highly susceptible cultivars were still dominant in Huanghuai Region, moderately resistant cultivars were identified for the first time, which indicated the improvement of breeding for FHB resistance in this region. It should be noted that the comparison is not very accurate due to the evaluations in different years and different locations. There are many factors affecting the severity of FHB by multi-location assessment, such as the year, region, wheat cultivars and environment. Xu et al. (2021) considered that weather conditions during the overwintering and subsequent grain colonization periods appeared to be more important than the preanthesis sporulation period in affecting the relative prevalence of *F. graminearum* and *F. asiaticum*. In addition, there was only one control cultivar when the two-year data were compared. For the evaluation of FHB resistance under multiple environmental conditions, there are relatively few factors involved in this study. Further studies should increase the number of identification sites and control cultivars to reduce the experimental error, and improve the accuracy of the experiment for this pathosystem that is highly dependent on environment (location/years). However, this result confirms the trend of change of FHB resistance of cultivars over the 4 years briefly.

Deoxynivalenol was the most frequent and abundant *Fusarium* mycotoxin detected in wheat cultivars from Hefei and Yangzhou, which is in agreement with the report of Cui et al. (2013) and Xu et al. (2019). They collected 506 wheat samples from Jiangsu and Anhui and DON was the most predominant mycotoxin detected in all samples. Juodeikiene et al. (2011) also reported that DON was a good marker of *Fusarium*-infected. In the current study, the DON accumulation was significantly correlated with disease index in Yangzhou. Cultivars from MLYR accumulated the least DON, follow by UYR, while the DON content in NH and SH was significantly higher (Figure 5A). This is consistent with a previous study by Yan et al. (2020). In Nanping, the situation is distinct from other locations, as NIV was the predominant mycotoxin. This is in agreement with the predominant rice cultivation and lack of wheat in this region, NIV producing *F. asiaticum* was mainly identified in Nanping (Zhang et al., 2012; Yang et al., 2018). Because of the different types of trichothecene, it is difficult to compare the resistance to mycotoxin accumulation between 2015 and 2019. So, in the case of evaluation of mycotoxin accumulation resistance by natural infection, it is necessary to consider the chemotype of local *Fusarium* populations. Wheat cultivars are also under the pressure of the local *Fusarium* population. It is reported that DON producing FHB pathogens dominated most wheat producing areas including Huanghuai (SH and NH) and the middle and lower reaches of Yangtze River Region (MLYR), but NIV producers were predominant in the upper valley of Yangtze River Region (UYR; Zhang et al., 2012; Yang et al., 2018). Evaluation of resistance to mycotoxin accumulation is not included in the national wheat registration program now. In recent years, breeders started to select cultivars with resistant to mycotoxin, but generally only for DON, and did not consider the differences of the dominant mycotoxins in different regions. Therefore, we suggest that the chemotype of *Fusarium* populations in both wheat originating and evaluating region should be considered when estimating the resistance to mycotoxin accumulation.

Breeding for FHB resistance is a long-term task, different ecological environments and wheat cultivars both have significant effects on the DI and toxin accumulation of FHB. Wheat may be more susceptible to *Fusarium* infection under future climate environment conditions (Vaughan et al., 2016). In China, climate change as well as changes in farming systems allowed FHB to gradually spread to NH and SH regions in the past 10 years, where it is now a threat to the main wheat production area in China (Qiu et al., 2014). Field evaluations are important for determination of FHB resistance. In this study, we observed that resistance levels are influenced by regional weather. Extreme disease pressure, both high and low, may cause inaccurate evaluation. To reduce this effect, it is important to include multi-regional evaluations in the national wheat cultivar registration program in China. To improve the FHB resistance of cultivars, China National Crop Variety Approval Committee changed the requirements in the past 10 years. Highly susceptible cultivars cannot be registered in MLYR, where FHB epidemics are frequent. FHB resistant cultivars in Huanghuai Region (SH and NH) which is the main wheat producing area would have priority in the registration program. In this study, based on a national-scale evaluation, we found an improvement of FHB resistance in the past 4 years, revealing the new policy led to cultivars with a higher resistance level. In addition, although significant correlation between DON and DI was observed at the population level, for individual cultivars, the DON accumulation cannot be simply evaluated by FHB resistance (Figure 5) in both breeding procedure and national trials to avoid losing some of the low DON accumulation cultivars. Therefore, we suggest the evaluation of mycotoxin accumulation should be emphasized, especially the research and development of wheat cultivars that accumulate only low mycotoxin levels, which is to be included in breeding procedures and used as a reference in evaluation of national cultivar trials.

## Data Availability Statement

The original contributions presented in the study are included in the article/Supplementary Material; further inquiries can be directed to the corresponding authors.

## Author Contributions

ZY, HZ, and TGL performed the entire project together, collected the data, performed the data analysis, and wrote the manuscript. WQC and JF supervised the project. TL, CW, and AD participated in manuscript revision. All authors contributed to the article and approved the submitted version.

## Funding

This work was supported by the National Key R&D Program of China (2017YFE0126700), and the National Natural Science Foundation of China (32172379), and China Agriculture Research System (CARS-03), and the Agricultural Science and Technology Innovation Program of the Chinese Academy of Agricultural Sciences (CAAS-ASTIP, CAAS-ZDRW202002).

## Conflict of Interest

The authors declare that the research was conducted in the absence of any commercial or financial relationships that could be construed as a potential conflict of interest.

## Publisher’s Note

All claims expressed in this article are solely those of the authors and do not necessarily represent those of their affiliated organizations, or those of the publisher, the editors and the reviewers. Any product that may be evaluated in this article, or claim that may be made by its manufacturer, is not guaranteed or endorsed by the publisher.
