# Supplementary material for: Evaluation of Fusarium Head Blight Resistance in 410 Chinese Wheat Cultivars Selected for Their Climate Conditions and Ecological Niche Using Natural Infection Across Three Distinct Experimental Sites
Source: Front Plant Sci. 2022 May 25;13:916282. doi: 10.3389/fpls.2022.916282 (PMC9195592; doi:10.3389/fpls.2022.916282)
Supplement: Supplementary file 3 [file Data_Sheet_1.docx]

Supplementary Material

# Supplementary Figures

**Figures S1-S2**

**
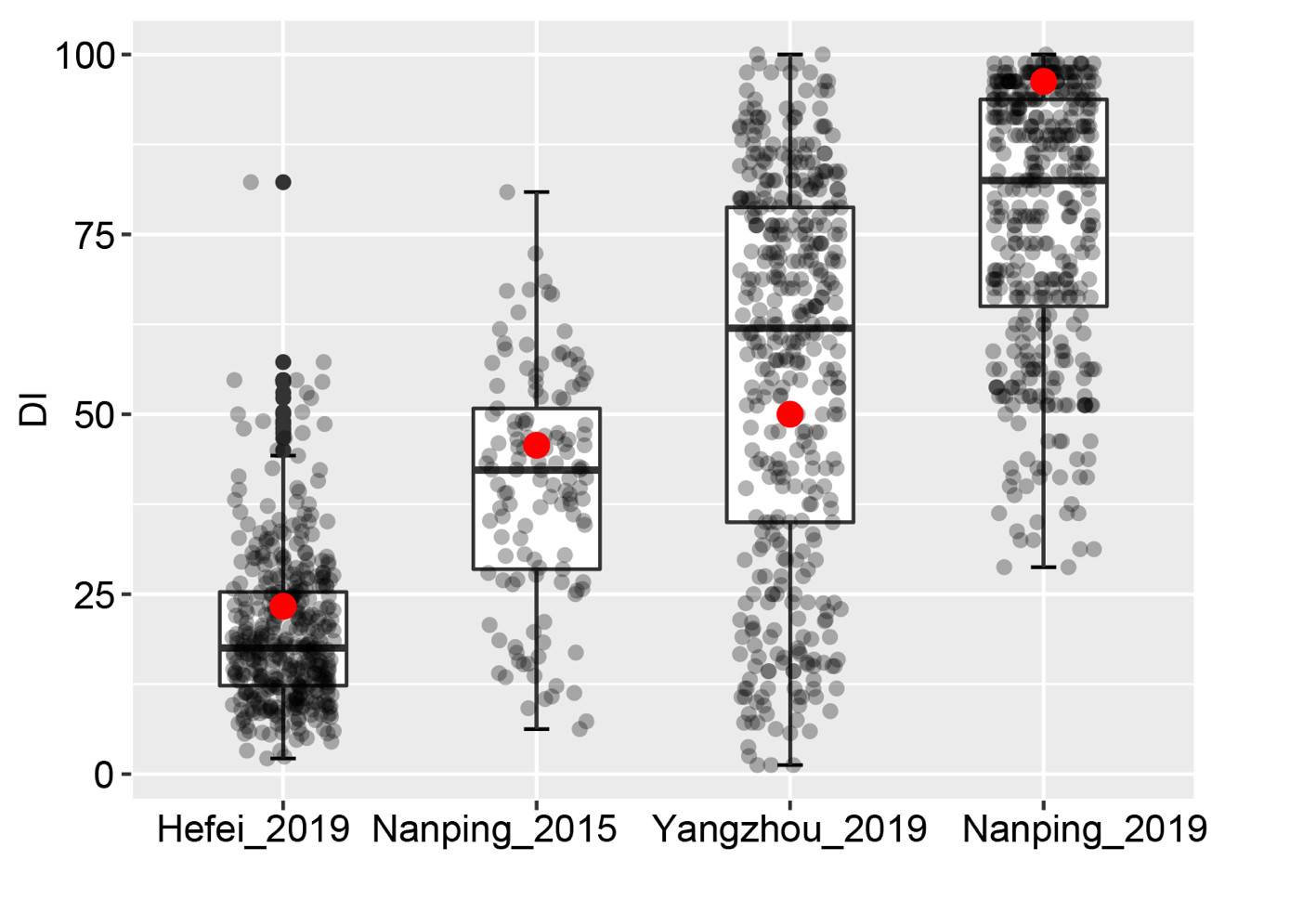
**

# Figure S1. Disease indexes of the control cultivars Zhoumai in the three regions in 2019 and the

# Nanping in 2015. The red dots indicate the DI of cultivar Zhoumai 18 in the 2015 and the three

# 2019 trials.


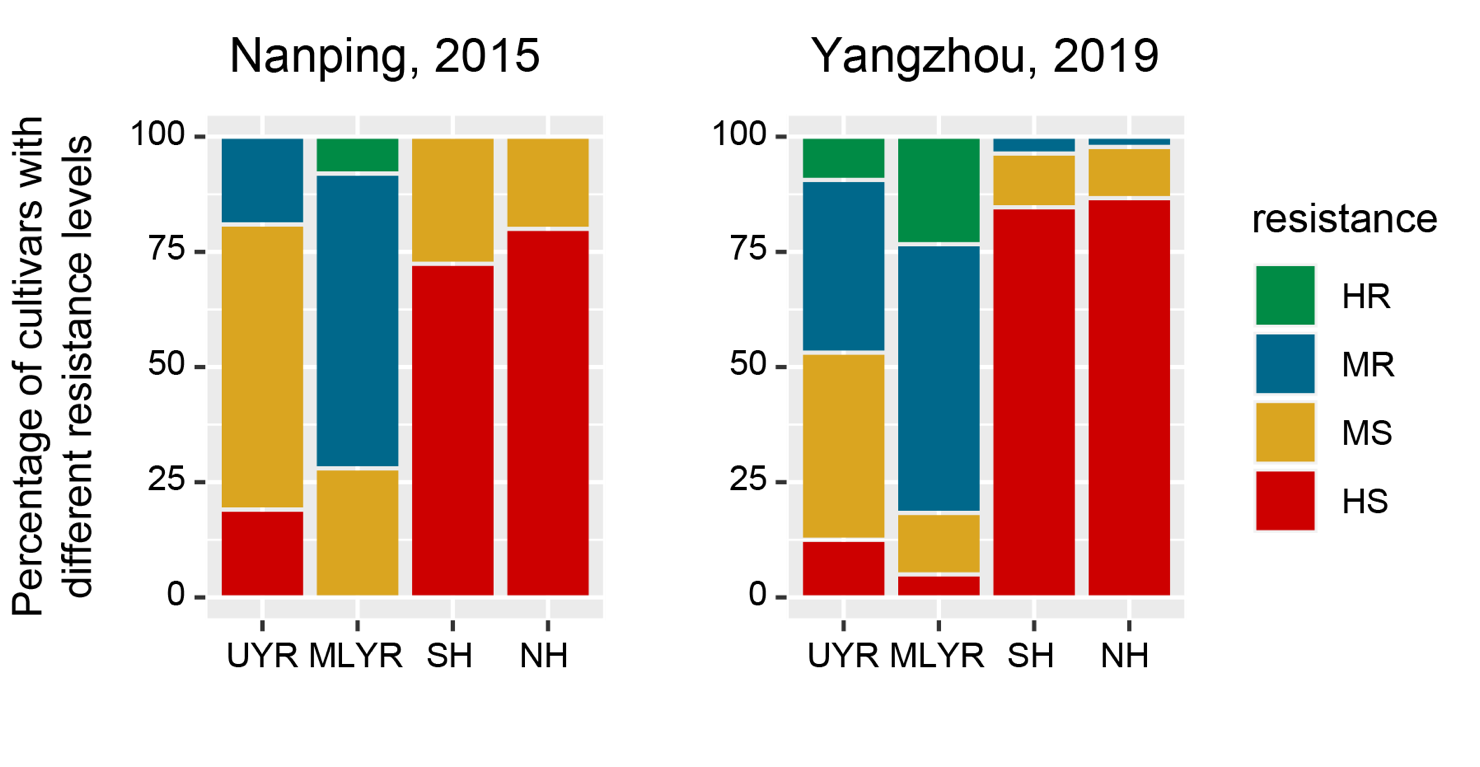


**Figure S2.** **Comparison of resistant levels to FHB based on fixed DI values between 2015 and 2019. Cultivars from all four ecological zones showed increased FHB resistance in 2019.**
